# Impact of Different Daily Light Integrals and Carbon Dioxide Concentrations on the Growth, Morphology, and Production Efficiency of Tomato Seedlings

**DOI:** 10.3389/fpls.2021.615853

**Published:** 2021-03-03

**Authors:** Brandon M. Huber, Frank J. Louws, Ricardo Hernández

**Affiliations:** Department of Horticultural Sciences, North Carolina State University, Raleigh, NC, United States

**Keywords:** carbon dioxide, PPFD, controlled environment agriculture, vertical farm, light-emitting diodes, energy consumption, production cost

## Abstract

Indoor growing systems with light-emitting diodes offer advantages for the growth of tomato seedlings through uniform and optimized environmental conditions which increase consistency between plants and growing cycles. CO_2_ enrichment has been shown to improve the yield of crops. Thus, this research aimed to characterize the effects of varied light intensities and CO_2_ enrichment on the growth, morphology, and production efficiency of tomato seedlings in indoor growing systems. Four tomato cultivars, “Florida-47 R,” “Rebelski,” “Maxifort,” and “Shin Cheong Gang,” were subjected to three different daily light integrals (DLIs) of 6.5, 9.7, and 13 mol m^–2^ d^–1^ with a percent photon flux ratio of 40 blue:60 red and an end-of-day far-red treatment of 5 mmol m^–2^ d^–1^. The plants were also subjected to three different CO_2_ concentrations: 448 ± 32 (400-ambient), 1010 ± 45 (1000), and 1568 ± 129 (1600) μmol mol^–1^. Temperature was maintained at 24.3°C ± 0.48/16.8°C ± 1.1 (day/dark; 22.4°C average) and relative humidity at 52.56 ± 8.2%. Plant density was 1000 plants m^–2^ until canopy closure. Morphological measurements were conducted daily to observe the growth response over time. In addition, data was collected to quantify the effects of each treatment. The results showed increases in growth rate with increases in the DLI and CO_2_ concentration. In addition, CO_2_ enrichment to 1000–1600 μmol mol^–1^ increased the light use efficiency (g_DM_ mol^–1^_applied_) by 38–44%, and CO_2_ enrichment to 1600 μmol mol^–1^ did not result in any additional increase on shoot fresh mass, shoot dry mass, and stem extension. However, the net photosynthetic rate obtained with 1600 μmol mol^–1^ was 31 and 68% higher than those obtained with 1000 and 400 μmol mol^–1^, respectively. Furthermore, the comparison of the light and CO_2_ treatment combinations with the control (13 mol m^–2^ d^–1^–400CO_2_) revealed that the plants subjected to 6.5DLI–1600CO_2_, 9.7DLI–1000CO_2_, and 9.7DLI–1600CO_2_ treatment combinations exhibited the same growth rate as the control plants but with 25–50% less DLI. Furthermore, two treatment combinations (13.0DLI–1000CO_2_ and 13.0DLI–1600CO_2_) were associated with the consumption of comparable amount of energy but increased plant growth by 24–33%.

## Introduction

High-quality transplants include seedlings that are free of disease/pests, that are compact but have high fresh and dry masses, and that exhibit high uniformity in both morphology and development (Kozai, 2005; Kubota et al., 2008). Currently, tomato seedlings are commonly grown in greenhouses or high tunnels, but these systems are subject to fluctuations in external weather, seasonality and solar radiation and thus could lead to seedlings that exhibit low uniformity. Indoor controlled environment (indoor CE) systems that use light-emitting diodes (LEDs) as the sole source of light have several advantages over other controlled environments. For example, indoor CE systems exhibit higher control of all environmental conditions, including temperature, radiation, spectrum, CO_2_ concentration, air velocity, photoperiod, and vapor pressure deficit. In addition, spectral customization can enhance the biomass and growth of tomato seedlings (Hernández et al., 2016). When combined, these environmental components that are controlled in indoor CE systems can increase the resource and energy use efficiency of plants. In addition, indoor CE systems provide consistent plant quality independent of the weather and increase the spatial and temporal uniformity of the plants (Ohyama et al., 2000; Kozai, 2005; Kubota et al., 2008). Although these systems have a higher electrical energy use, the high planting density, and short production cycle make them economically feasible (Ohyama et al., 2003; Kozai, 2007, 2013; Kubota et al., 2008).

Tomato (*Solanum lycopersicum* Mill.) is widely grown around the world and is the second most valuable vegetable crop in the United States (U.S. Department of Agriculture, 2016). It is also one of the most consumed vegetables in the world and provides healthy nutrients and antioxidants (Shi and Le Maguer, 2000). In addition, the vast majority of the tomato seedlings grown are started in specialized nurseries and transplanted to greenhouses and the field (Lewis et al., 2014). Furthermore, the use of grafted tomato plants has become an essential cultivation strategy in many parts of the world (Singh et al., 2017). For example, grafted tomato plants represent a significant percentage of the total tomato plants grown in Netherlands (75%), France (50%), Japan (40%), Korea (25%), and Vietnam (33%; Singh et al., 2017), and millions of grafted transplants are used in the United States, Italy, and Spain (Singh et al., 2017). Grafted tomato plants are utilized to increase plant vigor and thus achieve longer production cycles (Oda, 1999; Khah et al., 2006; Yarsi, 2011) and to confer disease resistance in tomato crops grown (Kaskavalci et al., 2009; Louws et al., 2010; Rivard et al., 2010; McAvoy et al., 2012). However, the propagation of grafted tomato plants at a large scale is a challenging process because the environmental conditions have to be adjusted to produce two plants (rootstock and scion) at the same growth rate to ensure proper stem matching (Kubota et al., 2008; Hu et al., 2016). Therefore, tomato transplants, including grafted seedlings, are suitable for indoor CE systems (Ohyama et al., 2003; Kozai, 2005; Nanfelt, 2016).

The light environment needs to be optimized to ensure desirable growth and reduce electricity consumption in indoor CE systems. The effects of the light intensity or photosynthetic photon flux density (PPFD) on the growth of tomato seedlings (Fan et al., 2013; O’Carrigan et al., 2014) and mature fruiting plants (Dorais, 2003; Torres and Lopez, 2011; Hao et al., 2017) have been studied. In general, an increase in the PPFD or daily light integral (DLI) increases the biomass and flower developmental rate (Uzun, 2006; Fan et al., 2013; Gómez and Mitchell, 2015). For example, in a growth chamber study, Fan et al. (2013) found that the shoot dry mass of tomato seedlings increased by 230% when the DLI was increased from 2.2 to 23.0 mol m^–2^ d^–1^ and by 51% when the DLI was increased from 6.5 to 13.0 mol m^–2^ d^–1^. Similarly, the use of supplemental lighting in a greenhouse to increase the DLI by 5.1 mol m^–2^ d^–1^ increased the shoot dry mass of tomato seedlings by 200% (Gómez and Mitchell, 2015). Although increasing the DLI generally increases growth, it is important to provide an adequate DLI to increase the production efficacy (growth per kilowatt hour). Fan et al. (2013) found that the best light level for tomato transplants was 13.0 mol m^–2^ d^–1^ because increasing the DLI beyond 23.0 mol m^–2^ d^–1^ resulted in only slight increases in the dry mass and no increase in the photosynthetic rate (Fan et al., 2013). Similarly, in tomato seedlings, O’Carrigan et al. (2014) showed that increasing the DLI from 17 to 27 mol m^–2^ d^–1^ only increased the shoot dry mass by 7%.

CO_2_ is often supplemented in indoor CE systems (Kozai, 2018) and CO_2_ enrichment is inexpensive under low room air exchange (0.001–0.1 h^–1^), which is common for indoor CE systems (Ohyama and Kozai, 1998). Although ambient CO_2_ levels (415 μmol mol^–1^) are acceptable for plant growth, enrichment is often necessary in indoor CE systems because a fully developed canopy can decrease the CO_2_ level to less than 200 μmol mol^–1^ (Bauerle, 1984; Both et al., 2017). CO_2_ enrichment to levels higher than the ambient conditions increases the yield and fruit quality of mature tomato plants (Calvert and Slack, 1975; Enoch et al., 1976; Nilsen et al., 1983; Fierro et al., 1994; Reinert et al., 1997; Li et al., 2007b; Khan et al., 2013; Mamatha et al., 2014; Pan et al., 2019). Although many studies have focused on the benefits of individual factors (light or CO_2_), fewer studies have highlighted the beneficial interaction of supplemental light and CO_2_ enrichment (Labeke and Dambre, 1998; Naing et al., 2016; Ting et al., 2017; Pan et al., 2019). Furthermore, prior studies have also suggested that high CO_2_ levels could partially compensate for a lower PPFD through comparable growth and dry mass (Mortensen and Moe, 1983) by increasing the net photosynthetic rate (Bencze et al., 2011; Ting et al., 2017).

Research reports have shown an increase in net photosynthetic rate and growth at CO_2_ enrichment concentrations of 700–900 μmol mol^–1^ and suggested that higher concentrations provide little improvement in growth (Behboudian and Lai, 1994; Fierro et al., 1994; Mamatha et al., 2014; Zheng et al., 2019). However, we hypothesize that CO_2_ enrichment above recommended values will have a significant positive impact in tomato seedlings’ net photosynthetic rate, growth, morphology due to: (1) seedlings are on the exponential growth stage with no competition; (2) the seedlings have a short growing period; and (3) CO_2_ enrichment is provided under relatively low DLI. Seedlings under optimal growing conditions show exponential growth. Once plants increase in size and plant competition is evident (canopy closure), the exponential growth phase changes to linear growth (Kirschbaum, 2011). During the exponential growth phase, plants show greater responses to high CO_2_ concentrations (Monje and Bugbee, 1998; Lewis et al., 2002). In the present study, tomato seedlings are in the exponential growth phase with no plant-to-plant competition. Research has also demonstrated long term adaptation to elevated CO_2_ concentration, including photosynthetic acclimation and leaf anatomy changes (i.e., lower stomatal density and conductance; Ziska et al., 1995; Zheng et al., 2019). However, these changes often take several days to occur (Ziska et al., 1995). Tomato seedlings in the present study were grown for 16–18 days (to the grafting stage) and 10–11 days from cotyledon expansion, which reduces the time for long term anatomical adaptation and photosynthetic acclimation to high CO_2_. Studies have shown down-regulation of photosynthesis under elevated CO_2_ (Kirschbaum, 2011). Studies have also shown that the down-regulation of photosynthetic rate under high CO_2_ concentrations can be affected by the DLI of the previous day (Bunce and Sicher, 2003), where high DLI in the previous day has a down-regulation effect on the following day, while lower DLI on the previous day does not. Young tomato seedlings in the present study were grown under constant relatively low DLI and therefore the down-regulation effect of previous day is minimized.

In the present study, the first objective was to study the effects of CO_2_ enrichment with DLI level of 6.5, 9.7, or 13.0 mol m^–2^ d^–1^ (relatively low PPFD of 100–200 μmol m^–2^ s^–1^) on the production of tomato seedlings. The second objective was to determine whether CO_2_ enrichment can maintain desirable plant growth under reduced light levels while maintaining comparable energy consumption, production cost, and high-quality seedlings. The third objective was to determine whether CO_2_ enrichment can reduce the production time of tomato transplants through an increased growth rate. In addition, calculations of the costs associated with different DLI and CO_2_ combinations were also performed and compared.

## Materials and Methods

### Plant Material and Growing Conditions

Four tomato cultivars were selected for this study: (1) “Rebelski” (*Solanum lycopersicum*; De Ruiter Seeds, Bergschenhoek, Netherlands), a popular indeterminate variety used in high tunnels and greenhouses for fresh-market tomatoes; (2) “Florida-47 R” (*Solanum lycopersicum*; Seminis Vegetable Seeds, St. Louis, MO, United States), a determinate variety used for field production; (3) “Maxifort” (*Solanum lycopersicum* x *Solanum habrochaites*; De Ruiter Seeds, Bergschenhoek, Netherlands), a vigorous rootstock that confers resistance to multiple soil-borne pathogens; and (4) “Shin Cheong Gang” (*Solanum lycopersicum*; De Ruiter Seeds, Bergschenhoek, Netherlands), an option for growers needing plants with disease resistance specifically against Fusarium race 3 and bacterial wilt. The seeds were sown in trays of Grodan Kiem rockwool plugs (27 × 20 mm; Grodan, Delta, Canada), and one seed was planted per cell at a density of 1000 plants m^–2^. The seeds were covered with vermiculite, and the trays were sub-irrigated until full saturation. Following irrigation, the trays were placed into a germination chamber at 28°C under darkness. Once radical emergence was evident (24–48 h depending on the cultivar), the trays were moved into three growth chambers subjected to the respective treatments. The temperature was set to 24°C during the day and 16°C at night to obtain a daily temperature average of 22°C. The relative humidity (RH) was maintained at 50–55% in all the treatments. The temperature and RH were monitored and logged every minute (HOBO onset UX100-023, Onset Computer Corporation, Bourne, MA, United States) during the experiment, and a summary is presented in Table 1. Dehumidifiers were included in each chamber to help manage the humidity. The plants were watered manually by sub-irrigating the trays twice daily using a nutrient solution composed of 90 mg L^–1^ N, 47 mg L^–1^ P, 144 mg L^–1^ K, 160 mg L^–1^ Ca, 60 mg L^–1^ Mg, 113 mg L^–1^ S, 105 mg L^–1^ Cl, and micronutrients (Jensen and Malter, 1995). The EC and pH of the nutrient solution were recorded daily (Hanna Instruments, Limena, Italy; Table 1).

**TABLE 1:** Environmental parameters measured inside the growth chamber^*z*^.

Treatment	6.5DLI–400CO_2_	9.7DLI–400CO_2_	13.0DLI–400CO_2_	6.5DLI–1000CO_2_	9.7DLI–1000CO_2_	13.0DLI–1000CO_2_	6.5DLI–1600CO_2_	9.7DLI–1600CO_2_	13.0DLI–1600CO_2_
DLI^y^ (mol m^–2^ d^–1^)	6.5	9.7	13.0	6.5	9.7	13.0	6.5	9.7	13.0
PPFD (μmol m^–2^ s^–1^)	100 ± 7	149 ± 9	200 ± 14	100 ± 6	150 ± 11	199 ± 15	100 ± 7	150 ± 8	200 ± 14
EOD-FR (mmol m^–2^ d^–1^)	13.5 ± 10	12.6 ± 8	12.5 ± 8	13.9 ± 11	12.8 ± 10	13.9 ± 11	15.8 ± 12	14.5 ± 11	14.1 ± 11
CO_2_ (μmol mol^–1^)		448 ± 32			1010 ± 45			1568 ± 129	
Day temp °C		24.4 ± 0.5			24.3 ± 0.4			24.2 ± 0.5	
Night temp °C		16.8 ± 1			16.9 ± 1			16.9 ± 1	
Relative humidity (%)		50.7 ± 7			51.9 ± 9			55.1 ± 8	
Photoperiod (h)					18				
pH					6.2 ± 0.1				
EC (dS m^–1^)					1.9 ± 0.1				

*^z^Averages and standard deviations from the three repetitions. ^y^Daily light integral.*

### CO_2_ Treatments

Three separate growth chambers had different CO_2_ level set points of 448 ± 32 (400-ambient), 1010 ± 45 (1000), and 1568 ± 129 (1600) μmol mol^–1^. The chambers were of identical size (width of 2.4 m, depth of 1.2 m, and height of 2.1 m) and had identical controls. The CO_2_ level was logged every minute and monitored (Viasala GMW115, Vantaa, Finland) to maintain sufficient levels (Table 1). During the dark period, all the chambers were ventilated to return the CO_2_ concentration to the ambient level of ∼400 μmol mol^–1^. The chambers used for the treatments were randomized before each of the three repeated experiments.

### Light Quality and Intensity

**FIGURE 1:**
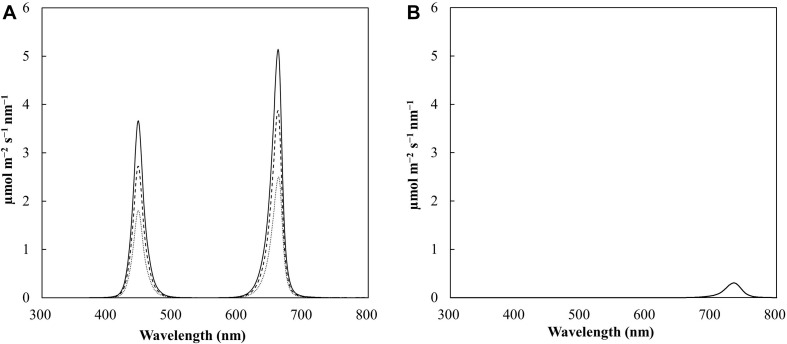
Spectral scan of the photoperiod at light intensities of 100 (dotted line), 150 (dashed line), and 200 (solid line) μmol m^– 2^ s^– 1^ **(A)** and spectral scan of the end of day (EOD) treatment (5 mmol m^– 2^ d^– 1^); **(B)** during the experiment. The data are averaged across the treatments. The photoperiod was 18 h (06:00-0:00), and the EOD-far-red (FR) treatment was delivered for 30 min (0:00-0:30).

Research has suggested that the best spectral quality for producing a tomato transplant is a blue:red ratio of 1:1 (Liu et al., 2011; Hernández et al., 2016). This ratio results in an increased photosynthetic rate (Kim et al., 2004; Li et al., 2007b; Liu et al., 2011), high plant compactness, and high fresh and dry masses (Hernández et al., 2016). However, some cultivars of tomato rootstocks have shown susceptibility to intumescence when grown under conditions lacking UV-B (Lang and Tibbitts, 1983; Craver, 2014). Further research has indicated that the inclusion of end-of-day far-red (EOD-FR) treatment combined with a spectrum of high blue PFD can be an effective strategy to mitigate intumescence (Eguchi et al., 2016). In addition, EOD-FR treatment also increases the hypocotyl length of tomato seedlings, which is desirable for tomato grafting (Chia and Kubota, 2010). With respect to EOD-FR treatment, research has shown that the saturation dose for hypocotyl extension and intumescence reduction is 5 mmol m^–2^ d^–1^, which can be achieved by exposure to 3.5 μmol m^–2^ s^–1^ for 24 min (Chia and Kubota, 2010; Eguchi et al., 2016). The LED fixtures used in this study (GE ARIZE, GEHL48HPPB1, GE Current, Boston, MA, United States) comprised a 42% blue (B) and 58% red (R) photon flux (PFD; close to the recommended 1B:1R ratio) with peaks at 448 nm (B) and 662 nm (R), both with a full width at half maximum (FWHM) value of 18 nm (Figure 1). Fixtures were installed inside each chamber to produce three light levels, namely, 100, 150, and 200 μmol m^–2^ s^–1^ PPFD, with an 18-h photoperiod.

The treatments within the chamber were separated from each other to ensure that the LED beam angle did not interfere with the other treatments. Light maps were produced for each treatment to ensure that the treatments exhibited minimal variation within each light intensity. The fixtures were set to an 18-h photoperiod (06:00–0:00) to provide three DLI treatments of 6.5, 9.7, and 13.0 mol m^–2^ d^–1^ for 100, 150, and 200 μmol m^–2^ s^–1^ PPFD, respectively (Table 1). EOD-FR treatment at 7.6 μmol m^–2^ s^–1^ PF with a peak at 737 nm with a FWHM of 29 nm (Phillips Greenpower LED Research Module Far Red, 929000632103, Amsterdam, Netherlands) was provided evenly to all the treatments for 30 min after the end of the photoperiod (0:00–0:30). The average EOD-FR dosage per treatment is presented in Table 1 and is above the saturation point. The photoperiodic and EOD lighting was measured with a spectroradiometer (PS-300, Apogee instruments, Logan, UT, United States) before and after each experimental run to measure the quality and quantity of PFD. The light measurements were averaged from eight locations from each treatment and are shown in Table 1. Height adjustable lights and growing tables were installed to maintain the same PFD at the top of the canopy throughout the experiment. To account for variations in the light gradient within a light treatment, the trays were systematically rotated daily.

### Measurement and Experimental Design

To track the growth of the stem diameter, total height, and leaf count, daily measurements were obtained from a subsample of 15 plants subjected to each treatment starting at day 10 until the final data collection. Commercially, tomato seedlings are typically spaced out (lower plant density) or grafted when plants reach canopy closure (∼3 true leaves and stem diameter of 1.8 mm). Therefore, a stem diameter of 1.8 mm was used as a threshold for data collection. Destructive data collections were performed when the last treatment reached a stem diameter of 1.8 mm. Averages from the subsamples of each experimental replication were obtained. The measurements included the stem diameter, hypocotyl length, epicotyl length, total height, leaf number, and fresh mass. The stem diameter was measured using a caliper (Mitutoyo Absolute Digimatic Caliper, Aurora, IL, United States), and the hypocotyl, epicotyl, and total heights were measured with a ruler. The number of leaves above a 1-cm threshold was counted, and the leaf area, including that of leaves greater than 1 cm, was also recorded using a leaf area meter (LI-3100, LI-COR Biosciences, Lincoln, NE, United States). Fresh samples were dried at 70°C and then weighed to record the dry mass.

The chlorophyll concentration was quantified as described by Moran and Porath (1980): two 56.6-mm^2^ leaf disks were cut from each plant of three subsamples per treatment per repetition. The gas exchange was measured at the end of the experiment using a portable photosynthesis machine (LI-6800, LI-COR Biosciences, Lincoln, NE, United States), and the results from three subsamples from each treatment were averaged. Measurements were performed on the youngest fully expanded leaf for all treatments and tomato cultivars. Environmental conditions for the measurements were 22°C, 60% RH and light levels and CO_2_ concentrations that matched the light and CO_2_ treatments (Table 1).

Statistical analyses were conducted to compare the treatments using JMP software 14.2 (SAS Institute, Cary, NC, United States). The experimental design was a split-plot design, the CO_2_ treatments were in different climate rooms and the three light levels were in each CO_2_ climate room. Light levels were randomized in each climate room for each of the three independent experiments. Also, climate rooms were randomized for each CO_2_ treatment at each of the three independent experiments. The treatment effects were run by cultivar and all cultivars had the same treatment response; therefore, the data for all cultivars was combined.

Linear regression was applied to the quantitative response to increasing the DLI at each CO_2_ concentration (all measured parameters) and to increasing CO_2_ for each DLI level (dry mass). To compare the slopes of the linear fit, a GLM procedure with Indicator Parameterization Estimates was used. Analysis of variance and mean separations via the Tukey-Kramer HSD test (alpha = 0.05) were computed when comparing the different CO_2_ treatments within each light level (Figure 3). Dunnett’s test was used to compare the treatments with different DLI and CO_2_ conditions to the 13.0DLI–400CO_2_ control treatment. The experiment was conducted three times.

### Evaluation of the Cost of Electrical Lighting and CO_2_ Enrichment

A summary of the variables, values, and units for the following calculations is shown in Table 2.

**TABLE 2:** Symbols, descriptions, values, and units used in the calculations.

Symbol	Description	Value	Unit
A	Total growing area	1	m^2^
APC	Areal electric power consumption	41.2–82.3	W m^–2^
B	Usage of CO_2_ per day	0.020–0.057	kg CO_2_ d^–1^
CC	Cost of CO_2_	0.58	$ kg^–1^
C_in_	CO_2_ concentration inside the chamber	0.0004–0.0016	mol mol^–1^
C_out_	CO_2_ concentration outside the chamber	0.0004	mol mol^–1^
D	Growing days per cycle	15–18	d
DEC	Daily electrical cost	0.07–0.13	$ d^–1^ m^–2^
DEN	Planting density	1000	plants m^–2^
E	Air exchange rate	0.10	h^–1^
E_r_	Electricity rate (United States)	0.09	$ kW h
K_m_	Volume to mass conversion for CO_2_ (22°C)	1.79	kg CO_2_ m^–3^
LPE	Lighting photon efficacy	3.0	μmol J^–1^
MF	Maintenance factor	0.90	–
N	Number of lamps	1.3–2.7	lamps m^−2^
P_n_	Net photosynthetic rate per LAI	0.001–0.003	kg m^–2^ h^–1^
P	Photoperiod	18	h
LAI	Leaf area index	1.9	m^2^ m^–2^
PPC	Per plant cost	0.0014–0.0026	$
PPFD	Photosynthetic photon flux density	100–200	μmol m^–2^ s^–1^
TCC	Total CO_2_ cost per production cycle	0.21–0.50	$ m^–2^
TEC	Total electricity cost per production cycle	1.13–2.26	$ m^–2^
UF	Utilization factor	0.90	–
V	Volume of growing facility	1	m^3^
WF	Wattage per fixture	30.5	W

The number of fixtures needed (N) to reach a set intensity can be described by Eq. (1) adapted from Aldrich and Bartock (1994), where PPFD is the desired PPFD (100, 150, or 200 μmol m^–2^ s^–1^ in our experiment), A is the total growing area (length × width; 1 m^2^ for ease of calculation), UF is the utilization factor [value that considers the beam angle distribution, growing area geometry, and reflectivity; 0.9 based on the information reported by Hernández and Kubota (2015)], MF is the maintenance factor [decrease in the fixture photon output over time; 0.9 based on the information reported by Hernández and Kubota (2015)], LPE is the lighting photon efficiency based on current technology (3.0 μmol J^–1^ or μmol W^–1^ s^–1^; GE current, Boston, MA, United States), and WF is the wattage required to power each fixture (30.5 W).

(1) $$\text{N}=\frac{\text{PPFD}\times \text{A}}{\text{LPE}\times \text{WF}\times \text{UF}\times \text{MF}}$$

The area electric power consumption (APC; W m^–2^) of lamps can be expressed by an Eq. (2) Hernández and Kubota (2015), which does not include the cost of HVAC cooling:

(2) $$\text{APC}=\frac{\text{N}\times \text{WF}}{\text{A}}$$

The daily electrical cost (DEC; $ d^–1^ m^–2^) can be expressed with Eq. (3), where APC (2) is multiplied by the photoperiod (P; h), divided by 1000 to convert from W h to kW h and then multiplied by the electricity rate Er ($ kWh^–1^), which varies by region. In this study, a rate of $0.09 was used based on the average in the United States (Lewis et al., 2014).

(3) $$\text{DEC}=\frac{\text{APC}\times \text{P}}{1000}\times \text{Er}$$

The final calculation was the total electricity cost (TEC; $ m^–2^), which is expressed by Eq. (4), where DEC is multiplied by the duration (D) to reach canopy closure and stem diameter of 1.8 mm (15–18 days depending on the cultivar and treatments used in this experiment).

(4) TEC=DEC×D

The usage of CO_2_ (B; kg CO_2_ d^–1^) can be described by Eq. (5; Ohyama and Kozai, 1998), where (P_n_) is the CO_2_ level per square meter of transplant growing area per hour (kg CO_2_ m^–2^ h^–1^), which was calculated based on the measured photosynthetic rate for each treatment combination using the youngest fully expanded leaf. The single leaf photosynthesis measurements (μmol m^–2^ s^–1^) were extrapolated to photosynthetic rate per meter square area using total leaf area per plant (19 cm^2^), plant density of 1000 plants m^–2^, photoperiod of 18 h, and the final LAI (Pn: 3.7–9.8 μmol m^–2^ s^–1^; LAI: 1.9). The other components of the equation include the following: K_m_ is the conversion factor from volume to mass for CO_2_ (1.79 kg CO_2_ m^–3^, at 22°C), E is the number of air exchanges per hour (0.1 h^–1^) which is considered an upper level exchange rate for enclosed controlled environments (0.001–0.1 h^–1^; Ohyama and Kozai, 1998), V is the volume of the growing area (1 m^3^ for ease of calculation), C_in_ is the desired setpoint CO_2_ concentration inside the facility, which varies between 400 and 1600 μmol mol^–1^ (0.0004–0.0016 mol mol^–1^) depending on the treatment, and C_out_ is the CO_2_ level outside the facility, which is typically near ambient levels (0.0004 mol mol^–1^). Pp represents the photoperiod (18 h).

(5) $$\text{B}=\left(\text{A}\times {\text{P}}_{\text{n}}+{\text{K}}_{\text{m}}\times \text{E}\times \text{V}\left({\text{C}}_{\text{in}}-C_{\text{out}}\right)\right)\times {\text{P}}_{\text{p}}$$

The total CO_2_ cost (TCC; $ m^–2^) is expressed by Eq. (6). As described in the equation, B (kg CO_2_ d^–1^, affected by the CO_2_ level, and PPFD) is multiplied by D (15–18 days depending on the treatments used in this experiment) and multiplied by the cost of CO_2_ (CC, $0.58 per kg, small volume price, Airgas, Radner, PA, United States).

(6) TCC=B×D×CC

The total production cost per square meter (PPC; $ m^–2^) can then be described by Eq. (7), which involves the addition of TEC ($ m^–2^; 4) and TCC ($ m^–2^; 6):

(7) PPC=TEC+TCC

## Results and Discussion

### Plant Growth (Fresh and Dry Masses and Net Photosynthetic Rate)

**FIGURE 2:**
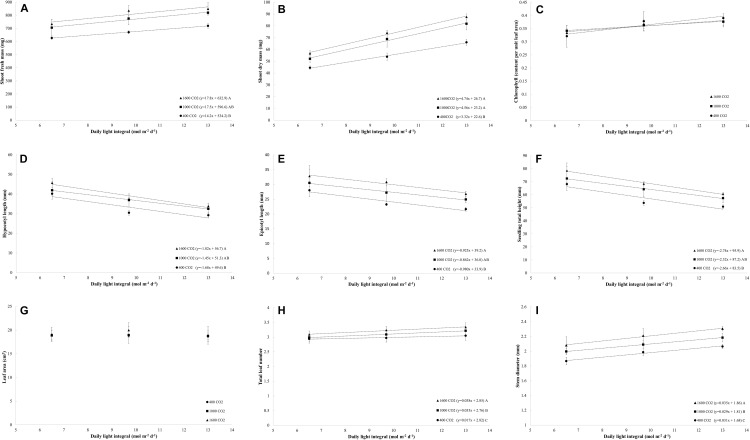
**(A–I)** Effects of the daily light integrals (DLIs: 6.5, 9.7, and 13 mol m^– 2^ d^– 1^) and CO_2_ levels (400, 1000, and 1600 μmol mol^– 1^) on the morphology, physiology, and growth rate of four cultivars of tomato (average of all cultivars) at day 18. The dotted lines represent significant linear regressions, and the equations are shown in parentheses. Different letters represent significant differences of the slopes.

For all light treatments, the fresh mass increased linearly with increases in the DLI for each level of CO_2_ (Figure 2A). CO_2_ enrichment to 1000 μmol mol^–1^ and 1600 μmol mol^–1^ resulted in the same rate of increase (slope) in the fresh mass as that obtained with increasing the DLI, and higher rates of increase in the fresh mass with increases in the DLI were observed under CO_2_-enriched conditions to 1600 μmol mol^–1^ than at 400 μmol mol^–1^ (Figure 2A). In general, under all CO_2_ treatments, the fresh mass increased by 16% with an increase in the DLI from 6.5 to 13.0 mol m^–2^ d^–1^. On average, under all DLI treatments, the fresh mass increased by 20% after CO_2_ enrichment to 1600 μmol mol^–1^ from 400 μmol mol^–1^. The combination of increasing the DLI from 6.5 mol m^–2^ d^–1^ to 13.0 mol m^–2^ d^–1^ and CO_2_ enrichment from 400 μmol mol^–1^ to 1600 μmol mol^–1^ increased the fresh mass by 36%.

Previous research studies on transplants have shown the impact of increasing the DLI on the fresh mass (Fan et al., 2013; Hernández and Kubota, 2014; Garcia and Lopez, 2020; Xu and Hernández, 2020). For example, Fan et al. (2013) showed a 27% increase in the fresh mass of tomato seedlings when the DLI was increased from 6.5 to 13.0 mol m^–2^ d^–1^ under LEDs (50B:50R, 12 h photoperiod) in a growth chamber. In a study of tomato, pepper, and cucumber transplants, Garcia and Lopez (2020) found fresh mass increases of 17, 33, and 18%, respectively, when the DLI was increased from 6.1 to 11.8 mol m^–2^ d^–1^ using supplemental high-pressure sodium (HPS) lighting in a greenhouse. In addition, Hernández and Kubota (2014) found a 28% increase in the fresh mass of cucumber seedlings when the DLI in a greenhouse was increased from 5.2 to 8.7 mol m^–2^ d^–1^ using supplemental LED lighting (0B:100R, 4B:96R, 16B:84R, and 18 h photoperiod).

The impacts of CO_2_ enrichment on the fresh mass of tomato seedlings have not been reported. However, many studies have demonstrated that CO_2_ enrichment increases the fresh fruit yield of tomato in a range of 19–124% when CO_2_ is increased to a range of 700–1400 μmol mol^–1^ separately from that of supplemental lighting (Calvert and Slack, 1975; Nilsen et al., 1983; Reinert et al., 1997; Khan et al., 2013; Mamatha et al., 2014; Pan et al., 2019).

The dry mass increased linearly with increases in the DLI, and this finding was obtained with all light treatments (Figure 2B). CO_2_ enrichment to 1000 μmol mol^–1^ and 1600 μmol mol^–1^ resulted in the same rate of increase (slope) in the dry mass as that obtained with increases in the DLI, and both CO_2_-enriched levels resulted in a higher rate of increase (slope) in the dry mass with increases in the DLI compared with that found with a CO_2_ concentration of 400 μmol mol^–1^ (slope; Figure 2B). Under all CO_2_ treatments, the dry mass increased by 53% when the DLI was increased from 6.5 to 13.0 mol m^–2^ d^–1^, whereas under all DLI treatments, the dry mass increased by 33% in response to enrichment to 1000–1600 μmol mol^–1^ from 400 μmol mol^–1^. The simultaneous increase in the DLI from 6.5 mol m^–2^ d^–1^ to 13.0 mol m^–2^ d^–1^ and CO_2_ enrichment from 400 μmol mol^–1^ to 1600 μmol mol^–1^ increased the dry mass by 165%.

**FIGURE 3:**
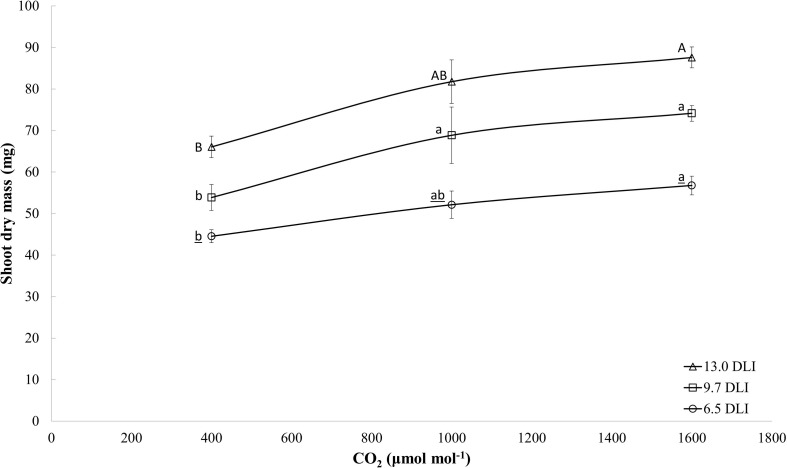
Effects of the CO_2_ enrichment treatments (400, 1000, 1600 μmol mol^– 1^) and light levels (DLIs: 6.5, 9.7, and 13 mol m^– 2^ d^– 1^) on dry mass of four cultivars of tomato (average of all cultivars) at day 18. The letters represent significant differences within each light level.

When comparing dry mass plant response to CO_2_ enrichment (all light levels combined), the dry mass increased linearly with increases in the CO_2_ level (*y* = 0.02x + 50.1; *R*^2^ = 0.29; and *p* = 0.0008). However, when analyzing the responses by DLI (Figure 3), a trend is present that at higher DLI levels, the dry mass response to CO_2_ is reaching a saturation point while at lower DLI the response is linear. Research studies have shown similar response (Bencze et al., 2011; Ting et al., 2017).

**FIGURE 4:**
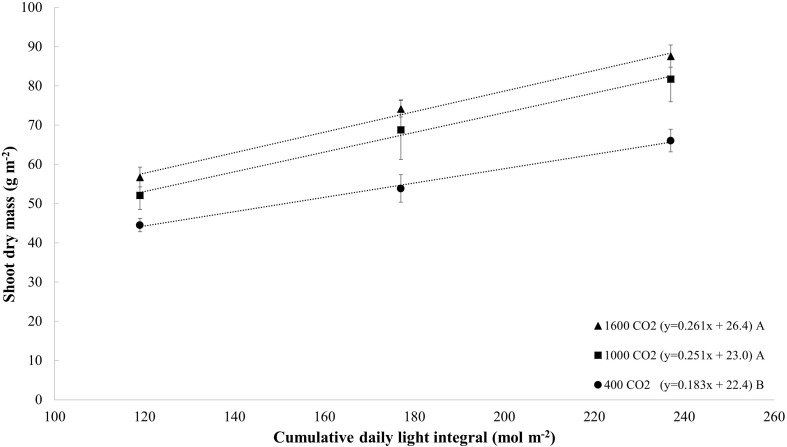
Dry mass (g m^– 2^) of tomato (all cultivars) grown with different cumulative photon flux (119, 177, and 237 mol m^– 2^) and CO_2_ levels (400, 1000, and 1600 μmol mol^– 1^). The dotted lines represent significant linear regressions, and the equations are shown in parentheses. Different letters indicate significant differences of the slopes.

The comparison of dry mass response per cumulative photon flux (Figure 4) showed that plants under CO_2_-enrichment (1000–1600 μmol mol^–1^) conditions produce 0.25–0.26 grams of dry mass per mole of light (g mol^–1^), whereas 0.18 g mol^–1^ is obtained under ambient CO_2_ conditions, which indicates that CO_2_ enrichment results in a 38–44% increase in light efficiency (g mol^–1^).

Previous research on transplants have shown the impact of increasing the DLI on dry mass (Fierro et al., 1994; Pramuk and Runkle, 2005; Fan et al., 2013; Hernández and Kubota, 2014; Pan et al., 2019; Garcia and Lopez, 2020; Xu and Hernández, 2020). In tomato seedlings, Fan et al. (2013) found a dry mass increase of 51% when the DLI was increased from 6.5 to 13.0 mol m^–2^ d^–1^ under LEDs (50B:50R, 12 h photoperiod). In greenhouses, Hernández and Kubota (2014) found a 47% increase in the cucumber seedling dry mass when the DLI was increased from 5.2 to 8.7 mol m^–2^ d^–1^ under LEDs (0B:100R, 4B:96R, 16B:84R, and 18 h photoperiod). In addition, Garcia and Lopez (2020) found an increase in the dry masses of tomato, pepper, and cucumber transplants ranging from 107 to 183% when the DLI was increased from 6.1 to 11.8 mol m^–2^ d^–1^ using HPS lighting in a greenhouse.

Previous research on tomato has also shown the impact of CO_2_ enrichment on the dry mass (Behboudian and Lai, 1994; Fierro et al., 1994; Li et al., 2007a; Wang et al., 2009; Pan et al., 2019). For example, studies have focused on the impact of increasing the CO_2_ concentration from an ambient level to 700–800 μmol mol^–1^, and this enrichment results in an increase in the dry mass of tomato plants of 27% when grown under florescent lamps (135 PPFD, 12 h photoperiod; Wang et al., 2009). A similar impact was shown under ambient greenhouse conditions by increasing the CO_2_ concentration from an ambient level to 700–800 μmol mol^–1^ resulting in a 16–27% increase of dry mass (Behboudian and Lai, 1994; Pan et al., 2019).

**FIGURE 5:**
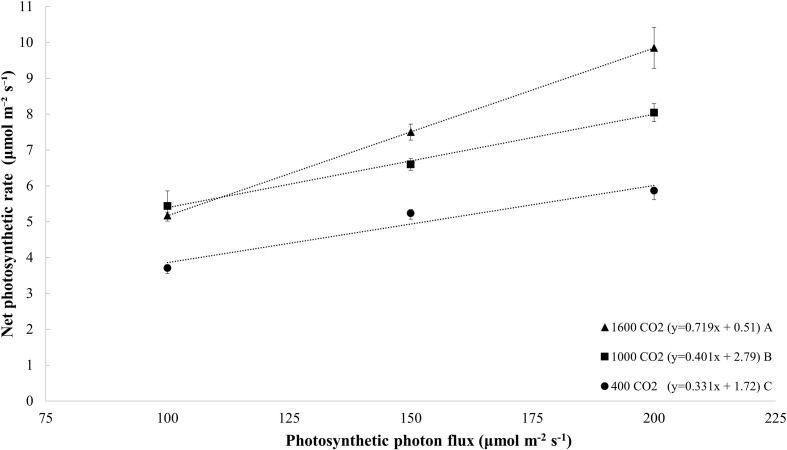
Net photosynthetic rate (μmol m^– 2^ s^– 1^) of tomato (all cultivars) at day 20 measured at different photosynthetic photon flux densities (100, 150, and 200 μmol m^– 2^ s^– 1^); and CO_2_ levels (400, 1000, and 1600 μmol mol^– 1^). The dotted lines represent significant linear fit and the equations are shown in parentheses. Different letters indicate significant differences in the slopes.

The net photosynthetic rate increased linearly with increases in the PPFD, and this finding was obtained with all light treatments (*p* = 0.005; Figure 5). Similarly, the net photosynthetic rate also increased linearly with increases in the CO_2_ level (*y* = 0.002x + 4.24; *R*^2^ = 0.36; and *p* = 0.008; data not shown). Plants exposed to a CO_2_ concentration of 1000 μmol mol^–1^ exhibited a higher rate of increase in their photosynthetic rate per increase (slope) in the PPFD than plants grown at 400 μmol mol^–1^ (Figure 5). In addition, CO_2_ enrichment to 1600 μmol mol^–1^ was associated with a higher rate of increase in the photosynthetic rate per PPFD than those obtained with CO_2_ levels of 400 and 1000 μmol mol^–1^ (Figure 5). In general, under all CO_2_ treatments, the net photosynthesis increased by 66% with an increase in the PPFD from 100 to 200 μmol m^–2^ s^–1^. On average, under all PPFD levels, the photosynthesis rate increased by 52% in response to CO_2_ enrichment to 1600 μmol mol^–1^ from 400 μmol mol^–1^. The combination of increasing the PPFD from 100 μmol m^–2^ s^–1^ to 200 μmol m^–2^ s^–1^ and CO_2_ level from 400 μmol mol^–1^ to 1600 μmol mol^–1^ increased the net photosynthesis rate by 165%.

Previous studies with transplants have shown that increasing the PPFD and CO_2_ concentration increases the photosynthetic rate (Fan et al., 2013; Hernández and Kubota, 2014; Lanoue et al., 2018; Pan et al., 2019). For example, tomato seedlings exhibit an increase in their photosynthetic rate of 90% when the PPFD is increased from 150 to 300 μmol m^–2^ s^–1^ (50B:50R, 12 h photoperiod; Fan et al., 2013). In greenhouse tomato transplant production, Pan et al. (2019) found a 21–39% increase in the photosynthetic rate with an increase in the PPFD of 200 μmol m^–2^ s^–1^ with HPS lighting. With cucumber transplants, a 20% increase in the photosynthetic rate was observed when the PPFD was increased by 54 μmol m^–2^ s^–1^ in a greenhouse with supplemental LEDs (0B:100R, 4B:96R, 16B:84R, and 18 h photoperiod; Hernández and Kubota, 2014). In response to CO_2_ enrichment to 1000 μmol mol^–1^ from 400 μmol mol^–1^, Lanoue et al. (2018) found a 52% increase in the photosynthetic rate of tomato seedlings. Similarly, Pan et al. (2019) showed that the photosynthetic rate of tomato seedlings increased by 9–27% with the enrichment of CO_2_ to 800 μmol mol^–1^ from 400 μmol mol^–1^.

In the present study, increasing the PPFD and CO_2_ concentrations increased the net photosynthetic rate and consequently resulted in more growth (increases in fresh and dry mass). In general, the plant responses to increases in the PPFD follow a logarithmic curve: increases in photosynthesis are observed until a saturation point is reached (Lopez and Runkle, 2017; Eichhorn-Bilodeau et al., 2019), and after this light saturation point, photosynthesis no longer increases with increases in the PPFD due to limitations of the Calvin cycle (Lopez and Runkle, 2017), and more specifically the enzyme activity and concentration of Rubisco (Bjorkman, 1981; Sukenik et al., 1987; Rivkin, 1990; Orellana and Perry, 1992; Geider and McIntyre, 2002). At the seedling stage of tomato, the saturation point for PPFD has been reported to be approximately 1200 μmol m^2^ s^–1^; however, this saturation point is affected by other conditions, including the CO_2_ level (Ting et al., 2017). In our study of tomato seedlings, the PPFD levels used (100–200 μmol m^–2^ s^–1^) did not reach the light saturation point.

Similar to the PPFD, there is a saturation point regarding the benefits of CO_2_ enrichment on the net photosynthetic rate. Studies have indicated that the CO_2_ saturation point for tomato is approximately 1200–1500 μmol mol^–1^ at the late-seedling stage (Wang et al., 2013; Ting et al., 2017). For example, Ting et al. (2017) found that the maximum photosynthetic rate of tomato seedlings is 1500 μmol mol^–1^ with a PPFD of 600 μmol m^–2^ s^–1^; however, under a PPFD of 900 μmol m^–2^ s^–1^, a CO_2_ level of 1200 μmol mol^–1^ reached the photosynthetic rate threshold. In the present study, the net photosynthetic rate was not saturated at a PPFD of 200 μmol m^–2^ s^–1^ and a CO_2_ level of 1600 μmol mol^–1^, which suggested that the light intensity and CO_2_ concentration can be further increased to increase the photosynthetic rate. However, no additional increase in the dry mass was observed in the present study when the CO_2_ level was enriched above 1000 μmol mol^–1^. Since a similar leaf area was obtained with all light and CO_2_ treatments (same canopy light capture), an increase in the dry mass was expected with the increase in the net photosynthetic rate at a CO_2_ concentration of 1600 μmol mol^–1^. Several studies have reported a greater increase in leaf photosynthetic rate and a lower increase in plant dry mass with CO_2_ enrichment (Monje and Bugbee, 1998; Bunce and Sicher, 2003; Kirschbaum, 2011). A possible explanation is that the additional photoassimilates were partitioned to increase root growth or phytochemical biosynthesis, such as the biosynthesis of anthocyanin, and these effects were not quantified in the present study. Another possible explanation is that the increased amount of carbohydrates produced by higher photosynthetic rate could not be utilized by the plant (sink limitations) and are stored in the leaves as starch and sugars (Kirschbaum, 2011; Zheng et al., 2019).

Studies on seedlings have focused on the plant responses to either variations in the DLI or in the CO_2_ concentration independently, and fewer studies have investigated the responses of plants to variations in both environmental factors (Desjardins et al., 1990; Fierro et al., 1994; Pan et al., 2019). In the present study, an increase in the CO_2_ concentration to 1000–1600 μmol mol^–1^ from ambient conditions increased the light use efficiency (grams of dry mass per mole of light applied) by 38–44%; in addition, the comparison of the lowest light and CO_2_ treatment with the highest light and CO_2_ treatment showed an increase in the plant dry mass of 165% (Figures 2B, 4). Therefore, CO_2_ enrichment could be a strategy to increase the growth of young plants while reducing their energy consumption and production time (see section “Stem diameter and impact on production time”). Alternatively, CO_2_ enrichment to 1000–1600 μmol mol^–1^ and under the standard DLI (13 mol m^–2^ d^–1^) can also increase production efficiency by reducing production time and increase overall seedling growth in indoor growing systems.

The benefits of optimizing both the DLI and CO_2_ concentration have been shown in previous research, in which the DLIs were usually higher than 13 mol m^–2^ d^–1^. To our knowledge, the benefits of CO_2_ enrichment (greater than 1000 μmol mol^–1^) under lower DLIs (below 13 mol m^–2^ d^–1^) have not been previously assessed. Our study highlights the benefits of CO_2_ enrichment to high levels (1000–1600 μmol mol^–1^) under relatively low DLIs ranging from 6.5 to 13.0 mol m^–2^ d^–1^, and our findings highlight the potential of decreasing the light requirement of plants (25–50%) through CO_2_ enrichment without affecting the quality of the transplants.

### Chlorophyll Content

The chlorophyll content increased linearly with increases in the DLI, and this finding was obtained with all light treatments (Figure 2C). In general, the chlorophyll content per unit leaf area increased by 16% with an increase in the DLI from 6.5 to 13.0 mol m^–2^ d^–1^. However, in this study, an increase in the CO_2_ concentration did not affect the chlorophyll content per unit leaf area (Ct/leaf area; Figure 2C).

Research with transplants have shown that an increase in the DLI increases the chlorophyll concentration per leaf area (Hernández and Kubota, 2014; Pan et al., 2019). For example, in cucumber, Hernández and Kubota (2014) found a 27% increase in the chlorophyll concentration on a leaf area basis when the DLI was increased from 5.2 to 8.7 mol m^–2^ d^–1^. In addition, in tomato transplants, Pan et al. (2019) found a 41% increase in the chlorophyll content with an increase in the DLI of 2.9 mol m^–2^ d^–1^. This effect is generally attributed to an increase in the palisade rows found in thicker leaves with a lower specific leaf area as a result of a higher light intensity (Lichtenhaler et al., 1981), which allows for adaptation of the photosynthetic apparatus to capture more light when available (Boardman et al., 1975; Lichtenhaler et al., 1981).

Previous studies on CO_2_ enrichment have also reported a decrease in the chlorophyll content per leaf area with an increase in the CO_2_ concentration. For example, in tomato seedlings, CO_2_ enrichment to 700 μmol mol^–1^ and 1000 μmol mol^–1^ decreased the total chlorophyll content (Mamatha et al., 2014; Lanoue et al., 2018), whereas in rice and wheat, no increase in the chlorophyll content was observed after CO_2_ enrichment (Mulholland et al., 1997; Kim and You, 2010). In citrus, CO_2_ enrichment also reduced the chlorophyll content per unit leaf area by 5%, but this reduction under elevated CO_2_ was overcome by an increase in the total leaf number (Idso et al., 1996). This decrease in the chlorophyll content as a result of CO_2_ enrichment is generally explained by an increase in the starch content and the presence of enlarged starch granules in leaves, which is thought to decrease chloroplast structure and function and thus decrease chlorophyll production (Cave et al., 1981; Yelle et al., 1990).

### Plant Morphology

#### Hypocotyl Length, Epicotyl Length, and Seedling Total Height

The hypocotyl, epicotyl, and total heights of the seedlings decreased linearly with increases in the DLI, and this finding was obtained with all light treatments (Figures 2D–F). In addition, the hypocotyl, epicotyl, and total heights increased linearly with increases in the CO_2_ level (plant height: *y* = 0.01x + 54.2; *R*^2^ = 0.31; and *p* < 0.001; data not shown). The plants grown under CO_2_ concentrations of 1600 μmol mol^–1^ exhibited higher rates of increases (slope) in the hypocotyl, epicotyl, and total heights per increase in the DLI than those grown under a CO_2_ concentration of 400 μmol mol^–1^ (Figures 2D–F). In general, under all CO_2_ treatments, the hypocotyl, epicotyl, and total heights increased by 25–34% when the DLI increased from 6.5 to 13.0 mol m^–2^ d^–1^. On average, the hypocotyl, epicotyl, and total heights increased by 24% in response to CO_2_ enrichment to 1600 μmol mol^–1^ from 400 μmol mol^–1^.

Numerous studies have shown that increasing the DLI decreases the plant height, including the hypocotyl, epicotyl, and total heights (Fan et al., 2013; Hernández and Kubota, 2014; Gómez and Mitchell, 2015; Garcia and Lopez, 2020). For example, Garcia and Lopez (2020) found that the hypocotyl height of tomato decreased by 10% when the DLI was increased from 6.1 to 11.8 mol m^2^ d^–1^. Fan et al. (2013) found a 47% decrease in the total height of tomato seedlings when the DLI was increased from 2.2 to 13.0 mol m^–2^ d^–1^.

Previous studies have also shown that CO_2_ enrichment increases the overall height of transplants (Li et al., 2007b; Khan et al., 2013; Mamatha et al., 2014). For example, Li et al. (2007b) found a 22% increase in the plant height of tomato seedlings in response to CO_2_ enrichment to 700 μmol mol^–1^ from 360 μmol mol^–1^ CO_2_ in indoor systems. Similarly, a 54% increase in the total height of tomato seedlings was found after CO_2_ enrichment to 1000 μmol mol^–1^ from 400 μmol mol^–1^ (Lanoue et al., 2018). In greenhouse-grown tomato seedlings, Mamatha et al. (2014) found a 25% increase in the plant height after CO_2_ enrichment to 700 μmol mol^–1^ from 400 μmol mol^–1^. Similarly, Fan et al. (2013) found a 22% increase in the plant height in response to CO_2_ enrichment to 1000 μmol mol^–1^ from 400 μmol mol^–1^.

The decrease in plant height observed with increasing light intensity is an expected adaptive response of plants (Zhang et al., 2003). Low light intensities initiate shade-avoidance responses and increase stem extension to maximize light capture (Schmitt et al., 1999). Therefore, the increase in plant height triggered by increased light intensity in this study was expected. In addition, an increase in plant height with an increase in the CO_2_ level has been reported in the literature (Downton et al., 1990; Pushnik et al., 1995; Slafer and Rawson, 1997), and this effect is normally attributed to the increase in the growth rate leading to an overall larger plant (taller with a higher dry mass; Pritchard et al., 1999). However, in the present study, CO_2_ enrichment increased stem extension independently of the plant growth rate because the plant hypocotyl length and plant height were 26% higher at the same dry mass (Table 3). Therefore, the increase in the stem length obtained with CO_2_ enrichment can also be attributed to an increase in cell expansion due to cell wall loosening and cell water/solute uptake (Cosgrove, 1993; Ferris and Taylor, 1994; Taylor et al., 1994; Ranasinghe and Taylor, 1996; Cosgrove, 1997).

**TABLE 3:** Comparison of the plant physiological responses to different DLI and CO_2_ treatments with those obtained with the control treatment (13.0DLI–400CO_2_).

Physiological parameter	Control	6.5DLI 400CO_2_	9.7DLI 400CO_2_	6.5DLI 1000CO_2_	9.7DLI 1000CO_2_	13.0DLI 1000CO_2_	6.5DLI 1600CO_2_	9.7DLI 1600CO_2_	13.0DLI 1600CO_2_
Stem diameter (mm)	13.0DLI–400CO_2_	−10%	=	=	=	+6%	=	+7%	+12%
Hypocotyl length (mm)	13.0DLI–400CO_2_	+37%	=	+44%	+26%	=	+56%	+29%	=
Epicotyl length (mm)	13.0DLI–400CO_2_	+29%	=	+41%	=	=	+52%	+43%	=
Total height (mm)	13.0DLI–400CO_2_	+34%	=	+42%	+26%	=	+54%	+35	+19%
Leaf area (cm^2^)	13.0DLI–400CO_2_	=	=	=	=	=	=	=	=
Leaf number	13.0DLI–400CO_2_	=	=	=	=	=	=	=	+9%
Fresh mass (mg)	13.0DLI–400CO_2_	=	=	=	=	=	=	=	=
Dry mass (mg)	13.0DLI–400CO_2_	-33%	−18%	−21%	=	+24%	=	=	+33%
Chlorophyll per leaf area (g m^–2^)	13.0DLI–400CO_2_	-19%	=	−14%	=	=	−15%	=	=
Photosynthetic rate	13.0DLI–400CO_2_	−37%	=	=	=	+37%	=	+28%	+68%

*For the various treatments, the symbols “–” and “+” indicate whether the values obtained are lower or higher (and the corresponding percentages) than the value obtained with the 13.0DLI–400CO_2_ treatment, and the symbol “=” indicates that no difference was found between the respective treatment and the 13.0DLI–400CO_2_ treatment. The statistical analysis was based on comparison with the 13.0DLI–400CO_2_ (control) treatment and was performed using Dunnett’s method with p ≤ 0.05.*

The EOD-FR light used in this study increased hypocotyl, epicotyl, and total plant heights. Therefore, the plant heights obtained with all the treatments in this study would have been reduced if the EOD-FR treatment was not included. The application of EOD-FR has been shown to increase the hypocotyl length of tomato seedlings by 12–34% (Chia and Kubota, 2010) and is a strategy used to achieve a longer hypocotyl length to compensate for the excessive compactness caused by a high blue PF in the LED spectrum. However, based on the results of this study, high CO_2_ levels could eliminate the need for applying EOD-FR treatment if the only goal is to manage hypocotyl extension.

#### Leaf Area and Leaf Number

The total leaf area per plant was not affected by increases in the DLI or CO_2_ level (Figure 2G), and the leaf number marginally increased with increases in the DLI (*p* = 0.002; Figure 2H) and increased linearly with increases in the CO_2_ level (*y* = 0.0002x + 2.91; *R*^2^ = 0.56; and *p* < 0.001; data not shown). The plants grown under CO_2_ concentrations of 1600 μmol mol^–1^ exhibited the higher rate of increase (slope) in the leaf number with increases in the DLI, and this rate of increase was higher than that observed in the plants grown under a CO_2_ concentration of 1000 μmol mol^–1^ and 400 μmol mol^–1^ (Figure 2H). Similarly, plants in 1000 μmol mol^–1^ also showed a higher rate of increase (slope) then plants in 400 μmol mol^–1^ (Figure 2H). In general, under all CO_2_ treatments, the leaf number increased by 7% with an increase in the DLI from 6.5 to 13 mol m^–2^ d^–1^, and on average, under all DLI treatments, the leaf number increased by 8% in response to CO_2_ enrichment to 1600 μmol mol^–1^ from 400 μmol mol^–1^.

Although the leaf number was affected by CO_2_ enrichment and the DLI, the leaf area was not affected in this experiment. It was expected that plants grown under a lower DLI would increase their leaf area as a response to capture more light. Although the leaf area obtained with the different treatments was comparable, the treatments with higher CO_2_ and DLI resulted in a higher dry mass. Therefore, in this experiment, the increase in growth rate obtained with higher DLI and CO_2_ can be mainly attributed to a higher photosynthetic rate and not to an increase in the leaf area for enhanced light capture [see section “Plant growth (fresh and dry masses and net photosynthetic rate)”].

Vegetable transplant research has shown similar results where increases in the DLI had no impact on the leaf area (Currey and Lopez, 2013; Garcia and Lopez, 2020). For example, cucumber seedlings grown in a greenhouse showed no increase in leaf area when the DLI was increased from 6.1 to 11.8 mol m^–2^ d^–1^ using supplemental HPS lighting (Garcia and Lopez, 2020). Similarly, ornamental plugs in a greenhouse showed no increase in leaf area when the DLI was increased from 4.5 to 9.5 mol m^–2^ d^–1^ using supplemental LEDs (Currey and Lopez, 2013). However, there are conflictive results when comparing the response of leaf area specifically to CO_2_ enrichment. For example, Pritchard et al. (1999) reviewed 63 studies and found that 57% of the studies reported an increase in the leaf area with increase in the CO_2_ levels, whereas 10% of the studies showed a decrease in the leaf area, and the remaining 33% observed no effect.

Another possible explanation for the lack of differences in the leaf area between the treatments could be attributed to the EOD-FR treatment used in the present study. All the plants were exposed to a EOD-FR treatment based on the reported daily dosage (intensity × duration) required to maximize (90%) hypocotyl cell extension, which consequently will also increase leaf area (Chia and Kubota, 2010; Eguchi et al., 2016); therefore, it is plausible that leaf expansion was maximized by the EOD treatment in all DLI/CO_2_ treatments.

Previous studies with vegetable transplants have also shown an increase in the leaf number with increases in the DLI and CO_2_ level (Hernández and Kubota, 2014; Mamatha et al., 2014; Pan et al., 2019; Garcia and Lopez, 2020). For example, Hernández and Kubota (2014) showed an 11% increase in the leaf number of cucumber seedlings when the DLI was increased from 5.2–8.7 mol m^–2^ d^–1^. In tomato seedlings, Pan et al. (2019) found an increase of 12% in the leaf number in response to an increase in the DLI by 2.9 mol m^–2^ d^–1^ in a greenhouse. Similarly, Garcia and Lopez (2020) found a 16% increase in the leaf number of tomato seedlings when the DLI was increased from 6.1 to 11.8 mol m^–2^ d^–1^. In response to CO_2_ enrichment from 380 to 700 μmol mol^–1^, Mamatha et al. (2014) found a 24% increase in the leaf number of tomato plants. In addition, Pan et al. (2019) found an increase in the leaf number of 18% in a greenhouse in response to CO_2_ enrichment (800 μmol mol^–1^), but this increase was dependent on a sufficient DLI through supplemental lighting (>2.9 mol m^–2^ d^–1^). These increases in the leaf number observed with increases in the DLI and CO_2_ level can be explained by the increased growth rate.

#### Stem Diameter and Impact on Production Time

The stem diameter of tomato seedlings increased with increases in the DLI, and this effect was observed with all light treatments (*p* < 0.001; Figure 2I). Similarly, the stem diameter increased linearly with increases in the CO_2_ concentration (*y* = 0.0002x + 1.9; *R*^2^ = 0.55; and *p* < 0.001; data not shown). Plants grown under a CO_2_ concentration of 1600 μmol mol^–1^ exhibited a higher rate of increase (slope) in the stem diameter per increase in the DLI than those grown under CO_2_ concentrations of 400 and 1000 μmol mol^–1^; similarly, the plants grown under a CO_2_ concentration of 1000 μmol mol^–1^ exhibited higher rate of increase in the stem diameter as those grown with 400 μmol mol^–1^ CO_2_ (Figure 2I). In general, under all CO_2_ treatments, the stem diameter increased by 10% with an increase in the DLI from 6.5 to 13.0 mol m^–2^ d^–1^, and on average, under all DLI treatments, the stem diameter increased by 11% in response to CO_2_ enrichment to 1600 μmol mol^–1^ from 400 μmol mol^–1^. The combination of increasing the DLI from 6.5 mol m^–2^ d^–1^ to 13.0 mol m^–2^ d^–1^ and the CO_2_ concentration from 400 to 1600 μmol mol^–1^ increased the stem diameter by 24%.

**FIGURE 6:**
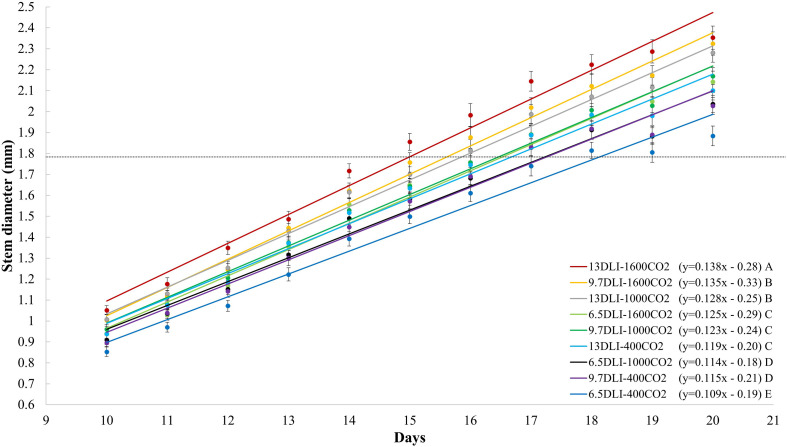
Daily stem diameter (mm) of tomato seedlings (all cultivars) subjected to treatments with daily light integrals (DLIs: 6.5, 9.7, and 13.0 mol m^– 2^ d^– 1^) and CO_2_ levels (400, 1000, and 1600 μmol mol^– 1^). Lines represent significant linear fit. The regression equation for each treatment is shown in parentheses. Different letters indicate significant differences in slope (rate of stem diameter increase).

**FIGURE 7:**
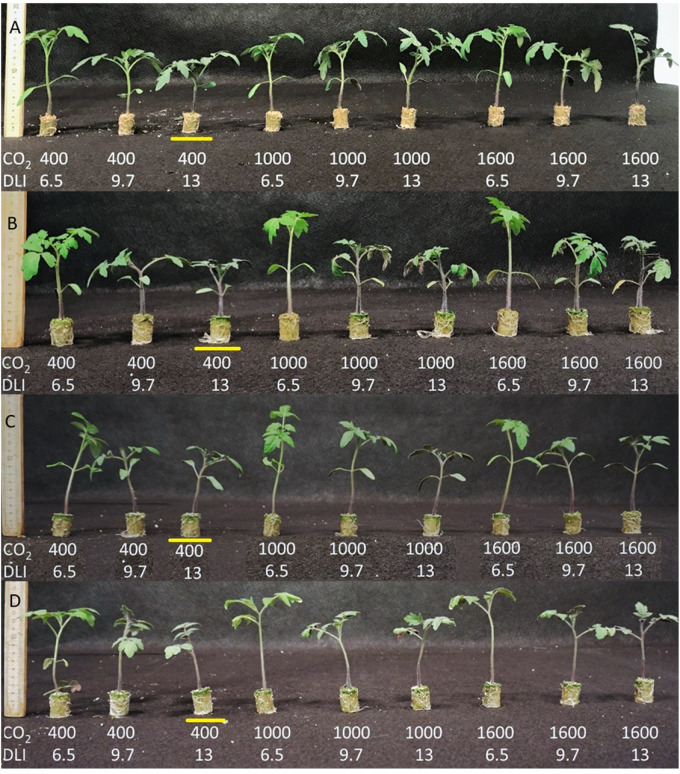
Seedlings of the tomato cultivars Florida 47 **(A)**, Shin Cheong Gang **(B)**, Rebelski **(C)**, and Maxifort **(D)** grown under various DLI and CO_2_ conditions. All the plants were harvested when the last DLI/CO_2_ treatment reached 1.8 mm (18–20 days). Commercial expected plant morphologies are shown in the control treatment 13DLI/400CO_2_ which are highlighted in the image for each cultivar.

At the seedling density used in this study (1000 plants m^–2^), the plants are grown until canopy closure and are then spaced to lower plant densities to prevent plant-to-plant competition and undesirable stretching. Several morphological factors serve as a threshold for reducing the plant density to prevent competition. For example, in tomato grafting, a stem diameter of 1.8 mm is often used as a threshold for both plant grafting and plant spacing. Therefore, the sooner the plant reaches this threshold, the shorter the production time. In the present study, the combination of different DLI and CO_2_ treatments affected the production time (time to reach 1.8 mm) of tomato seedlings (Figures 6, 7). The fastest growth rate was observed under the 13DLI–1600CO_2_ treatment, and these plants reached the threshold in a 12% shorter time than the control plants (Figure 6). The plants subjected to the 9.7DLI–1600CO_2_ and 13DLI–1000CO_2_ treatments reached the threshold in a 6% shorter time than the control plants (Figure 6). Comparable growth rates to the control plants were observed under the 6.5DLI–1600CO_2_ and 9.7DLI–1000CO_2_ treatments, and all of these plants needed 17 days to reach the threshold (Figure 6). The plants exposed to the 6.5DLI–1000CO_2_ and 9.7DLI–400CO_2_ treatments needed a 6% longer duration than the control plants to reach the threshold (Figure 6). These treatments with slower growth rates (6.5DLI–1000CO_2_, 9.7DLI–400CO_2_, and 6.5DLI–400CO_2_) also resulted in poor plant quality (lower shoot dry mass, smaller stem diameter, and lower chlorophyll content) and were deemed unsuitable growing conditions for transplants. The treatments that were superior or comparable to the control (13DLI–1600CO_2_, 9.7DLI–1600CO_2_, 13DLI–1000CO_2_, 6.5DLI–1600CO_2_, and 9.7DLI–1000CO_2_) provided suitable growing conditions for the production of high-quality tomato transplants. Based on our results, the tomato seedlings exposed to CO_2_-enriched concentrations of 1000 and 1600 μmol mol^–1^ reached the targeted stem diameter at the same time as the control plants (13 mol m^–2^ d^–1^, 400 μmol mol^–1^) despite 25–50% less light (6.5 to 9.7 mol m^–2^ d^–1^ DLI). Furthermore, using conditions consisting of CO_2_ enrichment to 1000–1600 μmol mol^–1^ and a DLI of 13 mol m^–2^ d^–1^, tomato seedlings can be produced at 6–12% faster rate than under the control conditions (Figure 6).

Studies have shown that increasing the DLI increases the stem diameter of transplants (Fan et al., 2013; Hernández and Kubota, 2014; Pan et al., 2019; Garcia and Lopez, 2020). For example, in indoor CEs, Fan et al. (2013) found a 16% increase in the stem diameter of tomato transplants when the DLI was increased from 2.2 to 13.0 mol m^–2^ d^–1^. Similarly, in cucumber transplants, Hernández and Kubota (2014) found a 20% increase in the stem diameter when the DLI was increased from 5.2 to 8.7 mol m^–2^ d^–1^, and in pepper transplants, Garcia and Lopez (2020) found a 20% increase in the stem diameter when the DLI was increased from 6.1 to 11.8 mol m^–2^ d^–1^.

CO_2_ enrichment studies have also shown an increase in the stem diameter of transplants (Egli et al., 1997; Li et al., 2007b; Khan et al., 2013). For example, in tomato transplants, Li et al. (2007b) found a 16% increase in the stem diameter in response to CO_2_ enrichment from 360 to 720 μmol mol^–1^. Similarly, in tomato, a 24% increase in the stem diameter was found after increasing the CO_2_ level from 400 to 1000 μmol mol^–1^ (Khan et al., 2013). Increases in the stem diameter have also shown benefits post-transplant. For example, an increased stem diameter of tomato transplants results in earlier yields (Liptay et al., 1981). Specifically, Liptay et al. (1981) found that transplants with stem diameters of 4.0–4.8 mm produced 32% more fruit at early harvest and thus exhibited a higher early yield than those with stem diameters of 3.2–4.0 mm. The observed increase in the stem diameter is expected and is explained by an overall increase in the plant biomass obtained under higher light (Grimstad, 1987; Dorais et al., 1991; McCall, 1992) and CO_2_ conditions (Bencze et al., 2011; Wang et al., 2013; Ting et al., 2017).

### Effect of Treatment Combinations on Plant Growth, Morphology and Sustainability

Table 3 presents the effects of the interaction of the DLI and CO_2_ level on plant growth and morphology. The combination treatments and their impact on plant growth and morphology were compared with standard growing conditions (13DLI–400CO_2_). Compared with the control (13DLI–400CO_2_) treatment, the 6.5DLI–400CO_2_ treatment, which involves 50% less light and the same CO_2_ level, produced a stretched plant (hypocotyl, epicotyl, and plant height) with a 33% lower dry mass and reduced values for the stem diameter, chlorophyll content, and photosynthetic rate (Table 3 and Figure 7). Decreasing the DLI from 13.0 to 9.7 mol m^–2^ d^–1^ while maintaining the same CO_2_ concentration (9.7DLI–400CO_2_) produced a plant with similar morphological characteristics (stem diameter, stem extension, and chlorophyll content) to the control plants with 25% less light but with a lower dry mass (18%), which was expected due to the reduction in the DLI (Table 3 and Figure 7).

Plants exposed to the 6.5DLI–1000CO_2_ treatment, which involves a 50% lower DLI and 150% higher CO_2_ level than the control treatment, produced stretched plants, and the total plant height was even greater than that of the plants under the 6.5DLI–400CO_2_ treatment, which highlights the contribution of CO_2_ enrichment on stem extension (Table 3 and Figure 7). The plants subjected to this treatment still showed a growth rate penalty (−21%) compared with the control plants, which was also attributed to the lower DLI.

The 9.7DLI–1000CO_2_ treatment, which involves a 25% lower DLI and 150% greater CO_2_ level than the control treatment, produced taller plants with no penalty in the growth rate (Table 3 and Figure 7). Even though the plants subjected to this treatment exhibited a higher plant height, this effect was solely due to a longer hypocotyl, which is likely affected by the EOD-FR treatment used in the experiment. The benefit of CO_2_ enrichment mitigated the impact of the reduced DLI, resulting in a similar growth rate. Therefore, this DLI and CO_2_ combination is suitable for reducing light requirements while maintaining plant quality to meet commercial standards.

The 13DLI–1000CO_2_ treatment, which involves the same DLI and a 150% greater CO_2_ level compared with the control treatment, resulted in plants with similar morphological characteristics as the control plants but an increased growth rate (24%), highlighting the benefits of CO_2_ enrichment on growth (Table 3 and Figure 7). Therefore, this DLI and CO_2_ combination is suitable for reducing the production time while maintaining plant quality to meet commercial standards.

The 6.5DLI–1600CO_2_ treatment, which consisted of a 50% lower DLI and a 300% greater CO_2_ level, produced taller plants with no penalty in the growth rate compared with the control treatment (Table 3 and Figure 7). The total plant height obtained with the 6.5DLI–1600CO_2_ treatment was greater than that obtained with the 6.5DLI–400CO_2_ and 6.5DLI–1000CO_2_ treatments, highlighting the contribution of CO_2_ enrichment to stem extension. Similar to the results obtained with the 9.7DLI–1000CO_2_ treatment, the benefit of CO_2_ enrichment mitigated the impact of the reduced DLI in the 6.5DLI–1600CO_2_ treatment, which resulted in a similar growth rate with half of the light. Even though the plants subjected to this treatment exhibited a higher plant height, the increased hypocotyl length is beneficial in the production of grafted plants. Therefore, this DLI and CO_2_ combination is suitable for reducing light requirements and maintaining plant quality to meet commercial standards.

The 9.7DLI–1600CO_2_ treatment, which involved a 25% lower DLI and a 300% greater CO_2_ level than the control treatment, produced taller plants with no penalty in the growth rate (Table 3 and Figure 7). The plants presented an increased plant height compared with the control plants, highlighting the contribution of CO_2_ enrichment to stem extension (Table 3 and Figure 7). Although the plants exposed to this treatment showed no penalty in the growth rate, an increase in the stem diameter was observed. Therefore, this DLI and CO_2_ combination is suitable for reducing both the light requirements and production time while maintaining plant quality to meet commercial standards. Moreover, the 13DLI–1600CO_2_ treatment, which consists of the same DLI and a 300% greater CO_2_ level than the control treatment, resulted in an increased total height with an increased growth rate (33%), highlighting the benefits of CO_2_ enrichment on growth (Table 3 and Figure 7). This increase in the total height is likely due to the increased growth rate and was not specific to the hypocotyl or epicotyl. Therefore, this DLI and CO_2_ combination is suitable for reducing the production time and increasing plant quality above commercial standards.

The reported impacts of CO_2_ enrichment on plant growth and morphology in this study utilize a spectrum (1B:1R) recommended for tomato transplant production based on previous research (Liu et al., 2011; Hernández et al., 2016) which optimizes photosynthetic rate (Kim et al., 2004; Li et al., 2007b; Liu et al., 2011), fresh and dry masses (Hernández et al., 2016), and produces a compact plant. Therefore, CO_2_ enrichment using other light spectrums during the photoperiod would be expected to impact the results due to altered growth rates, morphology, and photosynthetic rates. Furthermore, the use of EOD-FR also impacted this response. For example, the use of EOD-FR in our study contributed to 63% longer hypocotyl length (reduced excessive compactness) and reduced intumescence (preliminary study) making them commercially acceptable. Without the use of EOD-FR; however, plants for grafting would be commercially unacceptable due to compact internodes using the current spectrum.

Though the four cultivars used in this study showed no interaction of growth or plant morphology, growth rate differences were observed for cultivar independently of those from light and CO_2_ as shown with tomato seedlings (Hu et al., 2015). For example, in our study “Shin Cheong Gang” reached a 1.8 mm stem diameter (grafting threshold) at day 16, whereas “Florida-47 R” required 18 days when grown under 13DLI–400CO_2_. In addition, at day 18 “Shin Cheong Gang” shoot dry mass was 52 mg, whereas “Florida-47 R” was 77 mg under the same environmental conditions highlighting the difference in biomass accumulation between cultivars. Therefore, growth rate and other plant morphological differences may be observed when different cultivars are used. In addition, the physiological disorder intumescence was cultivar specific in our study affecting only “Maxifort” with 39% symptomatic foliage, whereas “Florida-47 R,” “Rebelski,” and “Shin Cheong Gang” showed no symptoms. The susceptibility of interspecific tomato rootstocks such as “Maxifort” to intumescence was previously reported (Eguchi et al., 2016). Without the use of EOD-FR, intumescence severity may further impact plant growth and decrease plant quality of susceptible cultivars.

In addition to the impacts on plant growth and morphology, varying the light and CO_2_ levels also offers an opportunity to optimize sustainability. According to the United States Department of Agriculture, sustainability involves five different components: (1) efficient use of nonrenewable resources, (2) enhanced environmental quality, (3) sustained economic viability, (4) satisfactory human food and fiber needs, and (5) enhanced quality of life of producers. A comprehensive evaluation of the components was not performed in this study, but the results regarding the usage of energy and CO_2_ (sustainability components 1 and 2) and the overall cost of light and CO_2_ (sustainability component 3) are presented in Table 4. The calculation of CO_2_ usage utilized a room air exchange rate of 0.1 h^–1^, which is still a very low ventilation rate. Therefore, most of the CO_2_ provided is used for plant growth and is not released outside the growing environment.

**TABLE 4:** Growing time (15–18 days), calculated energy usage of light-emitting diodes (LEDs; efficacy of LEDs used for the calculation is 3.0 μmol J^–1^; kWh per growing cycle), estimated operational cost of energy required to power LEDs ($ m^–2^ per cycle), estimated total CO_2_ consumption (kg CO_2_ per cycle), estimated operational cost of CO_2_ consumption ($ m^–2^ per cycle), and total operational cost for light and CO_2_ per cycle per square meter of growing area.

Treatment	Days to 1.8 mm	Total energy for lighting kWh	Lighting cost $ m^–2^	Total CO_2_ kg CO_2_	CO_2_ cost $ m^–2^	Total cost $ m^–2^
6.5DLI–400CO_2_	18	13.33	$1.20	0.36	$0.21	$1.41
9.7DLI–400CO_2_	17	18.89	$1.70	0.48	$0.28	$1.98
13.0DLI–400CO_2_	17	25.19	$2.26	0.54	$0.31	$2.57
6.5DLI–1000CO_2_	17	12.59	$1.13	0.53	$0.31	$1.44
9.7DLI–1000CO_2_	17	18.89	$1.70	0.64	$0.37	$2.07
13.0DLI–1000CO_2_	16	23.70	$2.13	0.73	$0.42	$2.55
6.5DLI–1600CO_2_	17	12.59	$1.13	0.54	$0.32	$1.45
9.7DLI–1600CO_2_	16	17.78	$1.60	0.71	$0.41	$2.01
13.0DLI–1600CO_2_	15	22.22	$2.00	0.86	$0.50	$2.50

The total energy and the total CO_2_ consumed to meet the different DLI and CO_2_ conditions are provided per square meter and growing cycle for every treatment combination (Table 4). When considering energy usage, production cost, and plant quality (growth and morphology) combined, the 9.7DLI–1000CO_2_, 13DLI–1000CO_2_, 6.5DLI–1600CO_2_, 9.7DLI–1600CO_2_, and 13DLI–1600CO_2_ treatment combinations are suitable for improving the sustainability of the current production practices (13DLI–400CO_2_). For example, the 9.7DLI–1000CO_2_, 6.5DLI–1600CO_2_, and 9.7DLI–1600CO_2_ treatments resulted in reductions in the energy usage of 19, 44, and 22%, respectively, which led to reductions in the production cost of 19, 44, and 22%, respectively, compared with the control treatment. The treatments with the same DLI as the control but a higher level of CO_2_ (13DLI–1000CO_2_ and 13DLI–1600CO_2_) also resulted in a small reduction in the production cost (1–3%) while reducing production time (6–12%) and increasing plant growth (24–33%). The cost of CO_2_ enrichment to 1600 μmol mol^–1^ is minimal in contained systems (0.1 h^–1^). Although the most economical treatment per cycle might be desirable, growers may benefit in producing more cycles per year based on the 13DLI–1600CO_2_ treatment.

## Conclusion

Despite the use of DLIs below the commercial standards (13.0 mol m^–2^ d^–1^), CO_2_ enrichment showed benefits on the growth and morphology of tomato transplants and maintained plant quality. Due to CO_2_ enrichment, the DLI requirements for producing tomato transplants can be reduced by 25–50% without affecting plant quality, which would reduce the production costs by up to 44%. Although hypocotyl elongation was observed with the treatments consisted of a lower DLI, this morphological characteristic can be controlled by the light spectrum. Alternatively, if a bigger plant with lower production time is desired, then maintaining a DLI of 13.0 mol m^–2^ d^–1^, CO_2_ enrichment at 1600 μmol mol^–1^ can reduce the production time by 12% and produce plants with a similar morphology and reduce costs by 3%.

With the increase in the light efficacy of LED lights, it is now possible to increase the production efficacy and sustainability of indoor systems by environmental optimization. The present study details the responses of tomato plants to two environmental components (light and CO_2_) and highlights an opportunity to optimize production based on selected goals. For example, large-scale tomato production can be optimized based on one or several of the following priorities: increase plant growth, reduce the production time, obtain a desired plant architecture, reduce energy usage, and/or increase affordability.

Future studies should investigate the post-transplant acclimation of these plants to field conditions and should focus on optimizing other environmental conditions to further optimize controlled environment systems.

## Data Availability Statement

The raw data supporting the conclusions of this article will be made available by the authors, without undue reservation.

## Author Contributions

BH: study conception and design, acquisition of data, analysis and interpretation of data, and drafting of manuscript. FL: analysis and interpretation of data, drafting of manuscript, critical revision, and funding acquisition. RH: study conception and design, analysis and interpretation of data, drafting of manuscript, critical revision, and funding acquisition. All authors contributed to the article and approved the submitted version.

## Conflict of Interest

The authors declare that the research was conducted in the absence of any commercial or financial relationships that could be construed as a potential conflict of interest.
